# Migration of fish bones into abdominal para‐aortic tissue from the duodenum after leading to duodenal perforation: a case report

**DOI:** 10.1186/s12876-021-01662-3

**Published:** 2021-02-23

**Authors:** Rong Wang, Jinyan He, Zhengquan Chen, Kunming Wen

**Affiliations:** Department of Gastrointestinal Surgery, Affiliated Hospital of Zunyi Medical University, Zunyi, 563003 Guizhou P.R. China

**Keywords:** Fish bone, Foreign body, Duodenal perforation, Case report

## Abstract

**Background:**

Migration of fish bones into abdominal para-aortic tissue after penetrating the junction of 3rd and 4th part of duodenum is incredibly rare.

**Case presentation:**

A 68-year-old man was admitted to our hospital with persistent colic in the lower abdomen after eating fish two weeks ago. Abdominal computed tomography (CT) scan showed High density streaks along the anterior and lower edges of the 3rd part of duodenum with peripheral exudation and localized peritonitis. Esophagogastroduodenoscopy didn’t find foreign bodies and perforations in the digestive tract. Laparoscopic surgery and intraoperative endoscopy were made to detect foreign bodies and perforation site was found. After transition to open surgery, the fish bone was found in abdominal para-aortic tissue and removed without complications. Postoperative recovery is smooth, and the patient resumed normal diet and was discharged.

**Conclusions:**

It is difficult to choose a treatment plan for foreign bodies at the 3rd part of the duodenum, because it is difficult to judge the damage caused by the foreign body to the intestine and the positional relationship with the surrounding important organs. Conservative treatment or surgical treatment both have huge risks. The handling of this situation will extremely test the psychology, physical strength and professional experience of the surgeon.

## Background

Ingestion of foreign bodies is a relatively common clinical problem in emergency departments worldwide. Most ingested foreign bodies (80–90 %) pass spontaneously. However, approximately 10–20 % of foreign bodies require an endoscopic procedure for removal and < 1 % require surgery [1, 2]. Approximately 1500 deaths occur in the United States annually because of ingestion of foreign bodies. Foreign body ingestion occurs most commonly in children (80 %), with a peak incidence from 6 months to 3 years of age [3–5]. The remaining patients (20 %) are adults. Foreign body ingestion is associated with a high risk of complications because of the size or shape of the foreign body or the host’s comorbidity. By contrast, adult patients with mental or psychiatric disorders, alcoholism, and drug abuse may ingest foreign bodies with nonfood objects. There are many kinds of foreign bodies in the upper digestive tract, most of which occur accidentally in adults when eating, and the main types are fish bones, chicken bones and chyme balls [3, 6, 7]. Fish bone can lead to complications such as esophageal microperforation, esophageal microperforation with reginal pneumomediastinum, esophageal microperforation with mediastinitis and abscess, microabscess over retropharyngeal space[1] and so on. Most patients recovered after receiving conservative therapy and endoscopic management. In this study, we present a rare case of migration of fish bones into abdominal para-aortic tissue after penetrating the junction of 3rd and 4th part of duodenum. The fishbone was taken out through open surgery, and the patient was discharged without any complication.

## Case presentation

**Fig. 1:**
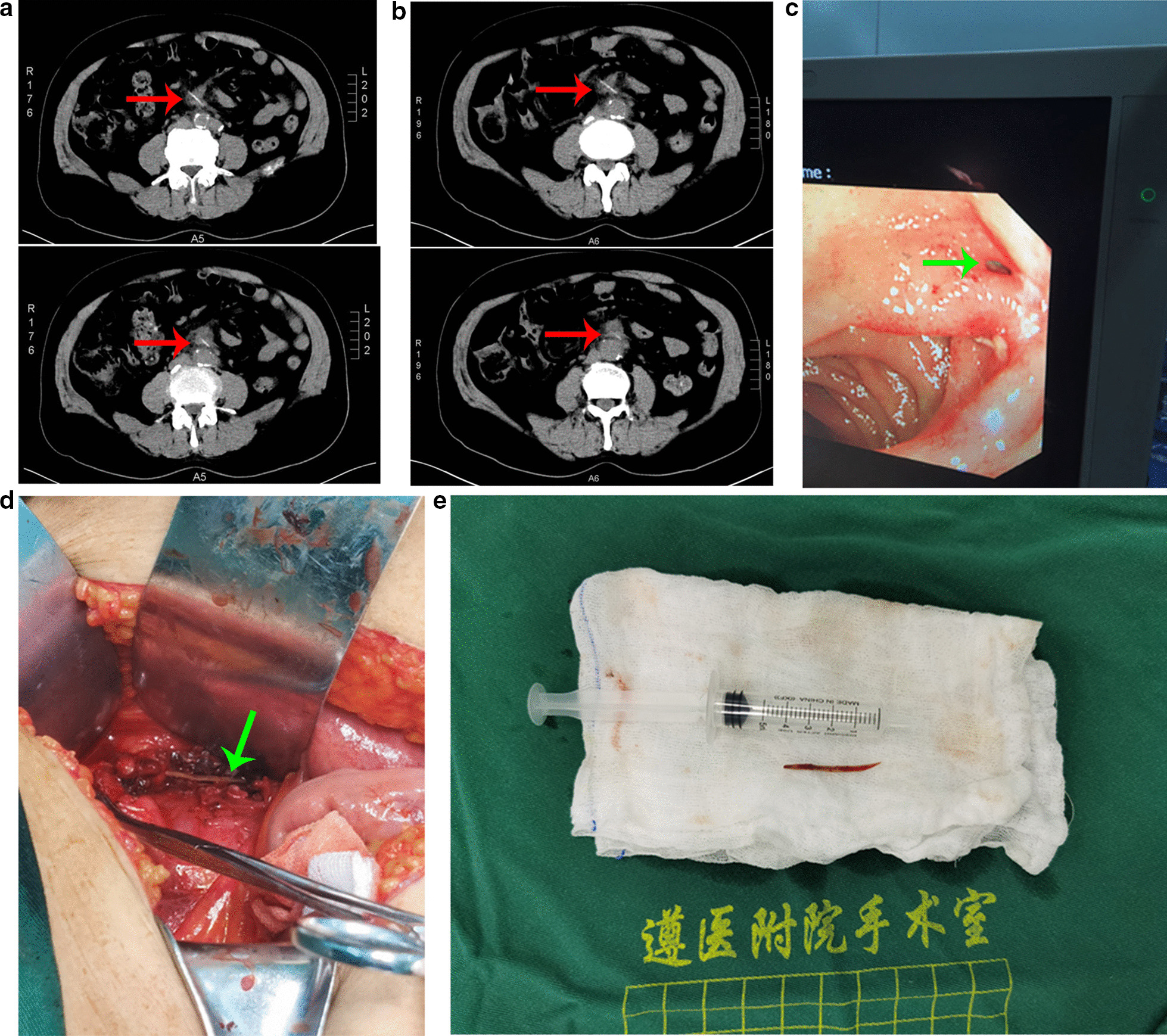
Migration of fish bones into abdominal para-aortic tissue from the duodenum after leading to duodenal perforation **a** Emergency abdominal computed tomography (CT) scan showed High density streaks along the horizontal anterior and lower edges of the duodenum. **b** Second abdominal computed tomography (CT) scan showed duodenal horizontal foreign body with peritonitis, local inclusional effusion or abscess. **c** Intraoperative endoscopy showed the foreign body had penetrated the intestinal wall at the junction between the horizontal and ascending parts of the duodenum. **d** A fishbone-like foreign body about 3 cm long was found in the inflamed tissue below the perforation. **e** The fishbone was like a knife

A 68-year-old man was admitted to our hospital with persistent colic in the lower abdomen which radiated to the back that had persisted for more than 10 days. He ate fish two weeks ago. There was no history of gastrointestinal bleeding or jaundice. Surgery for acute appendicitis and appendectomy was performed 6 years ago. The abdominal wall is palpated as slightly tense, with tenderness in the whole abdomen, significantly in the lower abdomen, no rebound pain and muscle tension. He visited the emergency department of our hospital and his emergency abdominal computed tomography (CT) scan showed high density streaks along the anterior lower edges of the 3rd part of duodenum with peripheral exudation and localized peritonitis (Fig. 1a). X-ray examination results showed no free gas under the diaphragm. Blood tests showed WBC 10.03 × 10^9^/L (normal range 3.5 × 10^9^/L –9.5 × 10^9^/L), N 0.68 (normal range 0.4–0.75), CRP 35.80 mg/L (normal range 0.068–8.2). The patient was diagnosed as “Foreign bodies in the duodenum” and hospitalized in department of Gastroenterology. Esophagogastroduodenoscopy (EGD) showed gastric body ulcer (locally with high-grade intraepithelial neoplasia); chronic atrophic gastritis with erosion (c-3) bile reflux; duodenal bulbous polyp. In order to observe the changes in the position of the foreign body, we recheck the abdominal CT. His second abdominal computed tomography (CT) scan showed foreign body in the 3rd part of duodenum with peritonitis, local inclusional effusion or abscess; Larger portion of the foreign body was located outside the intestine (Fig. 1b). The patient was transferred to gastrointestinal surgery for suspected duodenal foreign body and perforation. We gave conservative treatment measures such as fasting, drinking, and acid suppression. After one days of conservative treatment, the patient felt that the abdominal pain was relieved. On the second day of admission, we believed that the foreign body was clearly present and moving. Although the symptoms and signs were alleviated, the remained foreign body may cause perforation of the digestive tract or even shock and death, so we decided to operate. Then laparoscopy was made to detect and remove foreign body. The observation hole was located 5 cm below the umbilicus and was about 1 cm long. The main operation hole was 3 cm below the left midclavicular line and about 1.2 cm long. The remaining three operation holes were located 3 cm below the right midclavicular line and the intersection of the lateral third of the line connecting the bilateral anterior superior iliac spine and the umbilical cord, about 0.5 cm long. During the surgery, the retroperitoneum was opened to expose the 3rd and 4th part of the duodenum on both sides of the superior mesenteric vessels. No obvious inflammatory reaction was observed around the duodenum, and the location of foreign body penetration could not be determined. Considering colonoscopy is longer than gastroduodenoscopy, intraoperative colonoscopy were used and showed the perforation site between the 3rd and 4th part of the duodenum (Fig. 1c). Laparoscopy could not find the perforation site, and did not detect the abnormal area. So, we switched to open surgery. The median incision in the abdomen is about 20 cm long. Cut into the abdomen layer by layer, and continue to free the duodenum behind the upper mesenteric vessel on the right side. The lower wall of the intestine and the underside of the 3rd and 4th part of the duodenum showed heavy inflammation and tissue edema. The junction of 3rd and 4th part of the duodenum was partially perforated to a size of 0.5 cm. A fishbone-like foreign body about 3 cm long was found in the inflamed tissue below the perforation (in front of the right spine of the abdominal aorta) (Fig. 1d). We took out the foreign body and flushed the operation area. The duodenal decompression tube with a diameter of 4 mm is placed above the perforated part of the duodenum level and the jejunal nutrition tube were placed proximally and distally at 15 cm and 25 cm from the ligament of flexion, and two drainage tubes with a diameter of 7 mm were placed at the duodenal perforation. The operation lasts 6 h. Intraoperative vital signs were normal, with T 36.5 ℃, P 85 beats/min, R 20 times/min, BP 130/75 mmHg, SPO2 98 %. The bone was like a knife (Fig. 1f). Postoperative recovery is smooth, and the patient resumed normal diet and was discharged without any complication. The patient was hospitalized for 30 days. Enteral nutrition was started on the fifth day after the operation. The drainage tube and jejunal nutrition tube were removed after 3 weeks from the operation. The frequency of follow-up after discharge was once every two weeks. The last follow-up was one month after discharge, with normal nutritional status.

## Discussion and conclusion

Foreign bodies in the digestive tract are a global problem. Fishbone is a common foreign body in the digestive tract of adults. But perforation of a foreign body in the junction of 3rd and 4th part of duodenum is rare. In most cases, the fishbone were in the esophagus, stomach, and 2nd part of duodenum and could be excreted with feces or taken out through the mouth, which may also cause some complications, such as esophageal microperforation with reginal pneumomediastinum, mediastinitis and abscess [8], ileal perforation [9, 10], penetration through the posterior wall of the gastric antrum [11], cecal perforation [12], hepatic abscess secondary to gastric perforation [13–15], perforated acalculous cholecystitis [16], Pylephlebitis and Pyogenic Liver Abscesses [17–19], Small intestinal perforation [20, 21], aortic arch pseudoaneurysm [22], left subclavian artery-esophageal fistula [23]. But no reports of duodenal perforation have been reported. In this case, the fishbone migrated from the level of the duodenum to the front of the left spine of the abdominal aorta. It is very rare. If the abdominal aorta is penetrated further, it will cause serious consequences such as major bleeding, hemorrhagic shock and even death. So, the fishbone needs to be removed in time.

In the preoperative diagnosis, if there is a perforation, one of the diagnostic clues of duodenal perforation is the presence of retroperitoneal free air based on experience. The preoperative abdominal X-ray did not indicate any abnormalities in this case, which increased the difficulty of diagnosis. In contrast, Duarte et al. [24] reported that only 27% of the patients with perforated duodenal diverticulum showed retroperitoneal gas, and half of the patients had normal findings on plain abdominal X-ray film, suggesting that the diagnostic significance seems to be low. Abdominal CT scanning has been reported to be a useful diagnostic tool for perforated duodenum because of its ability to clearly display the retroperitoneal anatomy. In the present patient, abdominal CT showed duodenal foreign body, which is an important basis for our diagnosis.

In the treatment of this case, we mainly focus on three difficulties. One is the timing of the operation, the second is how to find the position of the fishbone and remove the fishbone smoothly, and the third is how to deal with duodenal perforation to avoid postoperative intestinal leakage.

Regarding the timing of surgery, it is difficult to judge before surgery. The patient has a history of accidental eating of fish bones, and there is a foreign body at the level of the duodenum according to his abdominal CT images. Because our gastroduodenoscopy cannot detect foreign bodies at the junction of 3rd and 4th part the duodenum, it is difficult to decide whether to choose surgery or conservative treatment. Although the patient’s abdominal pain was relieved, it seems that conservative treatment can be continued, the re-examination of the abdominal CT suspected that the foreign body was slightly displaced, indicating the possibility of perforation. At this time, the professionalism and courage of the surgeon are extremely tested. In principle, surgery is definitely the first choice of treatment, once the diagnosis of perforated duodenal diverticulum is made, except for some specific patients of advanced age and/or with severe underlying medical problems for whom there is an extremely high risk with surgical intervention. In order to avoid the serious consequences that may occur in the future caused by the foreign body penetrating the duodenum, such as major bleeding and shock, surgery was decided after consultation with the patient’s family. The postoperative results also confirmed that the surgery really needs to be done, and our decision is correct.

It has been reported that foreign bodies in the digestive tract most often appear in the esophagus and stomach and can be removed with a gastroduodenoscopy. There are also a few reports of foreign bodies in the duodenum. It was reported that chopstick and iron bar in 2nd part of the duodenum were taken out with upper-GI endoscopy [25, 26]. There was another case showed chopstick in 2nd part of the duodenum lead to duodenal perforation and emergency laparotomy with primary duodenorrhaphy [27]. But there is no literature on foreign bodies in the 3rd and 4th part of duodenum. Although gastroduodenoscopy has been developed for many years, the 3rd and 4th part of the duodenum is still difficult to see clearly. In order to remove foreign bodies, we can choose open surgery, laparoscopic surgery, or even endoscopy combined laparoscopic surgery. Consider reducing trauma and make full use of the advantages of laparoscopic vision, laparoscopic surgery was preferred. However, no obvious inflammatory reaction was observed around the duodenum, and the location of foreign body penetration could not be determined. In the previous literature, the doctors can find perforated foreign bodies and abscesses in the abdominal cavity and it is easier to determine the location of foreign bodies [27]. However, we did not detect foreign bodies and abscesses during the operation, which made it difficult to judge. So, we contacted and applied for multidisciplinary cooperation with department of Gastroenterology. The foreign bodies in the duodenum reported in other documents are all located in the descending part, so we encountered for the first time a foreign body in the duodenum that cannot be detected by a gastroduodenoscopy. We need a longer endoscope. An experienced endoscopist used a colonoscope and finally found perforation at the junction of the 3rd and 4th part of the duodenum. The foreign body has penetrated the duodenum and entered the abdominal cavity. It was obscured by the upper mesenteric vessel, and difficult to operate under laparoscopic surgery. We switched to open surgery. Along the inflammatory cord tissue, a fishbone-like foreign body about 3 cm long was finally found in the inflamed tissue below perforation (in front of the right spine of the abdominal aorta). There is a case report with duodenal occlusion by a large peach seed impacted in the duodenum [28]. The doctor used an endoscope to retract the peach seed into the stomach but could not take it out through the esophagus, and finally took out the foreign body from the stomach cavity using a laparoscope. There are no reports of laparoscopic surgery switching to open surgery to remove foreign bodies in the abdominal cavity. In this foreign body searching process, it can be described as difficult and easy to give up. It requires the patience, physical strength and skills of the surgeon.

According to previous experience, we took out the foreign body and flushed the operation area. According to the literature, if the integrity of the repair is in question, the right upper quadrant should be drained in anticipation of a duodenal fistula and a gastrostomy tube for drainage, and a jejunal feeding tube should be placed [29]. We used the four-tube method, including gastric tube, duodenal decompression tube, jejunal nutrition tube, and abdominal drainage tube. The duodenal decompression tube and the jejunal nutrition tube were placed proximally and distally at 15 cm and 25 cm from the ligament of flexion, and 2 drainage tubes were placed at the duodenal perforation. Common complications related to the repair of perforated duodenal diverticula have been reported to be duodenal fistula, intraabdominal abscess or sepsis-related complications, pancreatitis, and sustained wound infection. In the present case, the patient recovered smoothly without complications.

To summarize, it is difficult to choose a treatment plan for foreign bodies at the level of the duodenum, because it is difficult to judge the damage caused by the foreign body to the intestine and the positional relationship with the surrounding important organs. Conservative treatment or surgical treatment both have huge risks. The handling of this situation will extremely test the psychology, physical strength and professional experience of the surgeon.

## Data Availability

All data sets supporting the findings and inferences reported in this article are included within the article.

## Abbreviation

CT: Computed tomography

## References

[1] Lee CY, Kao BZ, Wu CS, Chen MY, Chien HY, Wu LW, Lin ST, Lai YH, Lin HJ (2019). Retrospective analysis of endoscopic management of foreign bodies in the upper gastrointestinal tract of adults. J Chin Med Assoc.

[2] Birk M, Bauerfeind P, Deprez PH, Hafner M, Hartmann D, Hassan C, Hucl T, Lesur G, Aabakken L, Meining A (2016). Removal of foreign bodies in the upper gastrointestinal tract in adults: European Society of Gastrointestinal Endoscopy (ESGE) Clinical Guideline. Endoscopy.

[3] Geng C, Li X, Luo R, Cai L, Lei X, Wang C (2017). Endoscopic management of foreign bodies in the upper gastrointestinal tract: a retrospective study of 1294 cases. Scand J Gastroenterol.

[4] Strickland M, Rosenfield D, Fecteau A (2014). Magnetic foreign body injuries: a large pediatric hospital experience. J Pediatr.

[5] Romine M, Ham PB, Yon JR, Pipkin WL, Howell CG, Hatley RM (2014). Multiple magnet ingestion in children. Am Surg.

[6] Zhang S, Cui Y, Gong X, Gu F, Chen M, Zhong B (2010). Endoscopic management of foreign bodies in the upper gastrointestinal tract in South China: a retrospective study of 561 cases. Dig Dis Sci.

[7] Lee HJ, Kim HS, Jeon J, Park SH, Lim SU, Jun CH, Park SY, Park CH, Choi SK, Rew JS (2016). Endoscopic foreign body removal in the upper gastrointestinal tract: risk factors predicting conversion to surgery. Surg Endosc.

[8] Shibuya H, Ikehara H, Andoh K, Horii T, Moriyama M, Yamao K, Gotoda T (2019). Endoscopic ultrasound-guided drainage of a mediastinal abscess caused by an ingested fish bone. Intern Med.

[9] Song J, Yang W, Zhu Y, Fang Y, Qiu J, Qiu J, Lin L, Wu W, Lin C, Wang Y (2020). Ingested a fish bone-induced ileal perforation: a case report. Med (Baltim).

[10] Zhao SG, Xu JJ, Xu L, Zheng JF, Zhou ZC, Jiang LQ (2019). Ileal perforation caused by a fish bone shortly after drug-eluting stent implantation for acute myocardial infarction. J Int Med Res.

[11] Zhang Z, Wang G, Gu Z, Qiu J, Wu C, Wu J, Huang W, Shen G, Qian Z (2019). Laparoscopic diagnosis and extraction of an ingested fish bone that penetrated the stomach: a case report. Med (Baltim).

[12] Kuwahara K, Mokuno Y, Matsubara H, Kaneko H, Shamoto M, Iyomasa S (2019). Development of an abdominal wall abscess caused by fish bone ingestion: a case report. J Med Case Rep.

[13] Venkatesan S, Falhammar H (2019). Pyogenic hepatic abscess secondary to gastric perforation caused by an ingested fish bone. Med J Aust.

[14] Yu W, Yu H, Ling J, Du J, Yin Z, Li C, Zhou M (2018). Hepatic Abscess Secondary to Stomach Perforation by a Fish Bone: a Rare Cause of Hepatic Abscess. Ann Hepatol.

[15] Mateus JE, Silva C, Beirao S, Pimentel J (2018). Hepatic Abscess Induced by Fish Bone Migration: Two Case Reports. Acta Med Port.

[16] Berevoescu NI, Grama FA, Welt L, Berevoescu M, Bordea A, Cristian DA (2019). An unexpected case of perforated Acalculous Cholecystitis caused by a fish bone. J Gastrointestin Liver Dis.

[17] Khandwala K, Ahmed A, Abid S (2019). Migration of fish bone into the portal vein resulting in Pylephlebitis and pyogenic liver abscesses. Am J Gastroenterol.

[18] Li J, Zhao D, Lei L, Zhang L, Yu Y, Chen Q (2019). Liver abscess caused by ingestion of fishbone: A case report. Med (Baltim).

[19] Queiroz RM, Filho FB (2019). Liver abscess due to fish bone ingestion. Pan Afr Med J.

[20] Taguchi T, Kitagawa H (2019). Fish bone perforation. N Engl J Med.

[21] Mora-Guzman I, Valdes de Anca A, Martin-Perez E (2019). Intra-abdominal abscess due to fish bone perforation of small bowel. Acta Chir Belg.

[22] Wang A, Zhou Y, Huang Q (2019). A fish bone induced aortic arch pseudoaneurysm in a male patient: a case report. Med (Baltim).

[23] Zhao S, Tinzin L, Deng W, Tong F, Shi Q, Zhou Y (2019). Sudden unexpected death due to left subclavian artery-esophageal Fistula caused by fish bone. J Forensic Sci.

[24] Duarte B, Nagy KK, Cintron J (1992). Perforated duodenal diverticulum. Br J Surg.

[25] Guo YN, Li F, Huang F, Yu T (2020). Endoscopic removal of a large foreign body retained in the duodenum: a case report. Med (Baltim).

[26] Wu C, Khan N, Yuan X, Ye L, Hu B (2018). Duodenal perforation caused by iron bar. Am J Gastroenterol.

[27] Li C, Yong CC, Encarnacion DD (2019). Duodenal perforation nine months after accidental foreign body ingestion, a case report. BMC Surg.

[28] de Filippo FR, Perrotta N, Cappiello A, Esposito T, Loffredo D (2013). Combined endo-laparoscopic approach in a patient with a duodenal foreign body and bowel obstruction. Updates Surg.

[29] Nirula R (2014). Gastroduodenal perforation. Surg Clin North Am.

